# EEG Patterns in Mild Cognitive Impairment (MCI) Patients

**DOI:** 10.2174/1874440000802010052

**Published:** 2008-08-12

**Authors:** Mary Baker, Kwaku Akrofi, Randolph Schiffer, Michael W. O’ Boyle

**Affiliations:** 1 Department of Electrical and Computer Engineering, Texas Tech University, USA; 2 Department of Neuropsychiatry, Texas Tech University Health Sciences Center, USA; 3 Department of Human Development and Family Studies, Texas Tech University, USA

## Abstract

An emerging clinical priority for the treatment of Alzheimer’s disease (AD) is the implementation of therapies at the earliest stages of disease onset. All AD patients pass through an intermediary stage of the disorder known as Mild Cognitive Impairment (MCI), but not all patients with MCI develop AD. By applying computer based signal processing and pattern recognition techniques to the electroencephalogram (EEG), we were able to classify AD patients versus controls with an accuracy rate of greater than 80%. We were also able to categorize MCI patients into two subgroups: those with EEG Beta power profiles resembling AD patients and those more like controls. We then used this brain-based classification to make predictions regarding those MCI patients most likely to progress to AD versus those who would not. Our classification algorithm correctly predicted the clinical status of 4 out of 6 MCI patients returning for 2 year clinical follow-up. While preliminary in nature, our results suggest that automated pattern recognition techniques applied to the EEG may be a useful clinical tool not only for classification of AD patients versus controls, but also for identifying those MCI patients most likely to progress to AD.

## INTRODUCTION

Mild Cognitive Impairment (MCI) refers to a syndrome in which a mild cognitive loss, typically in the domain of semantic memory, is measurable on neuropsychological test batteries, but is of insufficient strength to cause serious social, vocational or other functional impairment to the patient [1-3]. Interestingly, a large number of individuals with MCI progress to dementia as noted prospectively, perhaps as many as 15% per year [4].

Initial attempts at clinical intervention at the MCI phase of AD have produced mixed results [5-7]. Some studies suggest efficacy for currently available pharmacological therapies in treating MCI, but their impact is mostly transient. One possible explanation for this relative lack of success may be the heterogeneity of the MCI syndrome (e.g., amnesic versus non-amnesic MCI patients). In any case, treatment effects for MCI would undoubtedly be stronger if a subset of those patients most likely to progress to AD could be readily identified, thereby allowing for earlier treatment intervention.

Previous studies have suggested that signal features associated with the electroencephalogram (EEG) might be applicable to the problem, as several EEG abnormalities have been documented in the AD population. For example, [8] reports that EEG patterns in patients with AD typically show a shift towards heightened power in the lower EEG frequencies and reduced coherence of fast EEG rhythms. Note that EEG frequency is usually described in terms of five frequency bands: *delta* (below 4 Hz), *theta* (4 – 8 Hz), *alpha* (8 – 12 Hz), *beta* (12 – 22 Hz) and *gamma* (beyond 22 Hz)[9]. Most studies have not investigated the gamma bandwidth because of low power and higher susceptibility to noise, although a few such as [10] have studied EEG frequencies of up to 45Hz.

Other investigators have shown that unusual EEG patterns exist in AD patients and are characterized by a decrease in alpha and beta power, and a corresponding increase in delta and theta power as compared to normal age-matched controls [11-14]. Many investigators also hypothesize that the earliest modifications of the EEG occur in the beta and theta bands, while changes in the alpha and delta bandwidths appear later in the time course of the disease. However, this pattern is not universally found [15]. Notably, data regarding EEG changes in patients with MCI are less readily available, although significant differences between MCI subjects and controls have been noted in theta power as well as several other brain wave parameters [15-19]. For example, a study by Huang, *et al*. [20] using source localization techniques, suggested that anteroposterior localization of alpha power could potentially be a predictor of those MCI patients at greatest risk for AD progression.

EEG power is not the only brainwave characteristic that has shown differences between MCI patients, AD patients and age-matched controls. Coherence is another widely used measure in EEG-based AD/MCI studies. The former indexes the extent of synchrony between two cortical regions of the brain; the higher the coherence, the higher the synchrony. And, high synchrony is thought to reflect a functional linkage between the brain regions of interest. AD is believed to be a result of damage to functional cortical links in various brain areas [8, 21]. AD patients have been found to show reduced coherence for both resting and working EEG [8, 10, 21]. Some studies have found that MCI patients also show reduced coherence [10, 22].

Still other indicators of EEG synchrony include synchronization likelihood (SL) [23], cross mutual information (CMI) and Auto MI (AMI) [24], as well as global field synchronization (GFS) [25]. Studies using these parameters have suggested that AD and MCI patients generally show reduced synchrony as compared to healthy controls, and that AD patients show lower (less) synchrony than their MCI counterparts.

In light of the suggestion of Huang that antero-posterior localization of alpha power could potentially identify those MCI patients at greatest risk for AD progression, as well as other related findings in the literature, the present study was conducted to evaluate the EEG characteristics of MCI patients and to compare them to patients with AD, as well as neurologically normal, age-matched controls. We further sought to examine if it was possible to use an automated mathematical categorization technique (a k-means clustering algorithm) to sort MCI patients into subgroups based on their current EEG characteristics, specifically those who resembled AD patients versus those who did not, and to see if predictions could be made regarding their clinical status when assessed at a two-year follow-up examination.

## PARTICIPANT SELECTION

Participants were comprised of 17 AD patients (7 males, 10 females, mean age 75.07 years, 16 right-handed and 1 left-handed), 25 MCI patients (10 males, 15 females, mean age 75.36 years, all right-handed), and 16 age-matched controls (6 males and 10 females, mean age 75.53 years, all-right-handed), who volunteered to participate in the study as part of their involvement in the Memory Clinic at Texas Tech University Health Sciences Center (TTUHSC). All participants signed consent forms approved by the TTUHSC Internal Review Board (IRB) to participate in the study. One AD patient and 1 MCI patient were dropped from the study due to medication related issues.

## PROCEDURES

Standard EEG electrode placement (Jasper 10-20 electrode placement) was used for each participant and EEG data were recorded using a 16-channel longitudinal bipolar montage in the resting (eyes closed) condition. The bipolar montage was chosen because of its enhanced ability to record localized activity *via *its use of the neighboring electrode as a reference. The exact electrode sites employed in the study were Fp1, Fp2, F7, F3, F4, F8, T3, C3, C4, T4, T5, P3, P4, T6, O1 and O2. (See Fig. (**1**) for scalp locations and corresponding channel identification numbers; e.g., the Fp1–F3 channel is labeled Channel 1 in the diagram). The impedance of the electrode-skin interface was kept below 5kΩ at each electrode site. In addition to the EEG data, all participants were given complete physical and neurological examinations, neuropsychological testing, and whenever possible, an MRI scan.

All EEGs were recorded using a Medelev Valor system with a sampling rate of 256 Hz. EEG analyses were performed on 30 sec segments of artifact-free data, randomly selected from the raw EEG record. Data from each channel were filtered using a 0.5 to 50 hertz bandpass filter. The data segments were normalized by dividing the EEG data epoch for each channel by the total power present in that channel. This procedure is thought to eliminate differences between amplifier and impedance settings across channels and participants. These filtered and normalized data were then used in all statistical calculations.

## EEG ANALYSIS TECHNIQUES

Analyses were performed on the aforementioned EEG power data by applying a set of digital filters to divide the EEG signal into respective alpha, beta, delta and theta bandwidths. These analyses yielded average power estimates for each of the above frequency ranges, at each of the 16 EEG channel locations for each participant. EEG data for the control and AD groups were analyzed by performing pre-planned comparisons of average power for each channel within a given frequency bandwidth. A standard two-tailed t-test was used to determine statistical significance.

In all, three group comparisons were made (AD vs. controls, AD vs. MCI, and MCI vs. controls) for each of four frequency bands (delta, theta, alpha and beta) and for all 16 channels. Thus, the total number of comparisons was 192. Given the large number of comparisons made we elected to reduce our alpha level to p<.03 from the usual standard p<.05. While not a formal Bonferroni correction, this shift to a somewhat more conservative alpha accomplishes two desirable outcomes simultaneously: (1) it reduces the likelihood of a Type 1 error, and (2) at the same time it is not overly restrictive, allowing us to maximize the number of EEG features that could be used in the clustering process.

Channel locations and frequency bands showing significant differences between control and AD participants (henceforth referred to as the feature set) were subsequently used as inputs for a k-means clustering algorithm [26]. The k-means clustering algorithm assumes a user-specified number of classification categories (two in this case), and then calculates the centroid (mean EEG power) for each category (group or cluster). The value of the centroid is then iteratively adjusted until the differences between data points and the centroid for each group is minimized. Steps in the algorithm are as follows:

1. Choose *k* initial cluster centers. (In our case, *k* = 2.)
2. Calculate the Euclidean distances between the feature vectors (each participant is represented as a vector of EEG data) and the cluster centers. Assign every feature vector to the cluster center to which it is closest, thus creating *k* clusters.
3. Find the centroids of the newly-created *k* clusters.
4. Using the centroids of Step 3 as cluster centers, repeat Steps 2 and 3 until the centroids no longer change. At this point the algorithm is said to have attained *convergence,*i.e., they no longer change with subsequent iterations.

In general, the clusters generated by the k-means algorithm depend on the choice of initial cluster centers. It is therefore prudent to repeat the algorithm several times with different initial cluster center choices. Fortunately, the algorithm converges quickly, so running it several times is not very time consuming. Initial cluster centers can simply be chosen at random if there is no a-priori information regarding the data. However, there is often prior information, e.g., a rough idea of the numerical ranges of the feature vectors of each group or of the data as a whole. And, if the groups are well separated, the very same clusters will be generated for a wide range of initial center choices.

All t-tests and k-means clustering were conducted using MATLAB 6.5. We applied the MATLAB function *t-test a*nd our own k-means program algorithm (written in MATLAB code) to determine clusters.

In the first stage of the study, the aforementioned k-means algorithm was tested for its ability to segregate individuals into meaningful groups by attempting to categorize AD patients versus controls. In this regard, the algorithm proved particularly effective, achieving over 80% correct classifications. Subsequent to this initial test, in the second stage of the study, the same k-means algorithm was used to classify MCI patients into two subgroups; one resembling AD patients (subgroup 1) and one resembling controls (subgroup 2). In the final stage of the study, the output of the aforementioned subgroup classification was used to predict the status of individual MCI patients (i.e., progression to AD or not) at their two-year clinical follow-up examinations.

## FREQUENCY BAND POWER

Inter-group statistical comparisons of bandwidth power for AD and control participants revealed differences that were primarily restricted to the beta frequency range, where AD patients had significantly lower EEG power relative to controls in 12 of 16 channels. In fact, the only channels not differing between AD and controls in relative beta power were Channel 2 (F3-C3), Channel 4 (P3-O1), Channel 7 (C4-P4) and Channel 14 (F8-T4). It might be mentioned that in the delta frequency range, there was one channel (Channel 13) in which delta power was significantly greater in AD patients than controls [*t*(30) = 2.34; p<.02].

Further analyses of EEG power comparing MCI and control participants, revealed that 4 of the 16 channels showed significantly greater power in controls, all of which were found in the beta frequency range: Channel 2 (F3-C3) [*t*(38)=2.29; p<.02], Channel 6 (F4-C4) [*t*(38)=2.51; p<.01], Channel 8 (P4-O2) [*t*(38)=2.07; p<.03], and Channel 10 [*t*(38)=2.64; p<.01]. Note that EEG power in Channel 5 (FP2-F4) was the only location to show a significant difference between AD and MCI patients and this was found in the theta bandwidth [*t*(38)=2.53; p<.01] . While the physiological and behavioral importance of these differences in EEG power and location are not yet understood, their presence illustrates that even at rest there are verifiable differences between AD, MCI and controls in their respective EEG power profiles.

One focus of the present study was the attempt at categorizing MCI patients into two subgroups based upon their EEG profiles using the aforementioned k-means clustering technique. When employing this classification algorithm, 13 of the 24 MCI patients were categorized as members of subgroup 1 (more like AD patients), and 11 were categorized as members of subgroup 2 (more like control subjects). This classification was accomplished by using the 8 EEG features (locations) exhibiting the largest differences in relative beta power between AD patients and controls. These features included beta power differences found in Channels 1 (Fp1-F3), 8 (P4-O2), 9 (Fp1-T1), 10 (T1-T3), 11(T3-T5), 12 (T5-O1), 13(Fp2-T2), and 16 (T6-O2). As can be seen in Fig. (**2**), relative beta power values for MCI subgroup 1 are closely aligned with AD power values, while those for MCI subgroup 2 are more aligned with controls.

Of particular interest is the fact that for 6 patients returning for their two-year clinical follow-up examinations, the k-means clustering algorithm accurately predicted the clinical status of 4 patients. Specifically, there were 3 MCI patients that the algorithm predicted would not have progressed to AD, and indeed these individuals did not show any signs of AD at clinical follow-up. The algorithm also predicted that 3 other MCI patients would progress to AD however, only one of them had actually done so at follow-up. The later finding does not rule out eventual progression to AD in these two patients, but rather may be reflective of the disease progressing at a somewhat slower than anticipated rate.

## DISCUSSION

When relative power of the EEG as recorded from MCI patients was analyzed using an automated k-means clustering algorithm, it was discovered that two clearly identifiable subgroups emerged; one with EEG beta power profiles similar to AD patients (subgroup 1) and one more similar to controls (subgroup 2). The biological importance of this EEG power differential at various brain locations is not presently understood. But, it is intriguing to speculate that such differences may nonetheless be effective in distinguishing those MCI patients most likely to progress to AD from those less likely to do so, and our preliminary results suggest some promise for such an approach. It is particularly important to note that our predictions concerning the 3 MCI patients who were correctly identified as unlikely to progress to AD, and the 1 MCI patient who was accurately predicted to develop AD, were made without reference to any other diagnostic instruments and took place well before any clinical signs or behavioral manifestations of AD were actually exhibited in the MCI patients. Thus, our preliminary findings suggest that automated pattern recognition techniques applied to EEG brainwave characteristics, (particularly if supplemented by other physiological, genetic and neuropsychological measures), holds considerable promise for the early identification of those MCI patients most likely to progress to AD. Moreover, if our continuing evaluation of this diagnostic technique should prove reliable after testing a greater number of follow-up patients, it would have broad implications for the screening of participants in drug trials, and for providing guidance to patients and family members regarding their expectations for potential AD progression.

## Figures and Tables

**Fig. (1):**
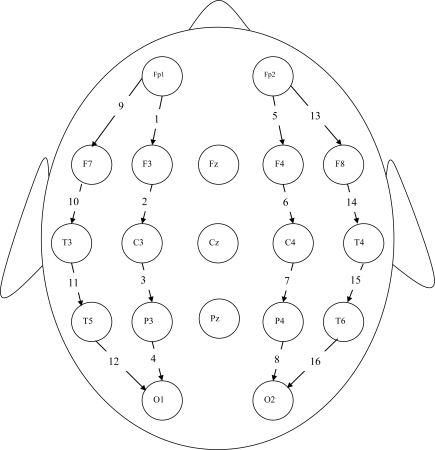
16-channel longitudinal bipolar montage

**Fig. (2):**
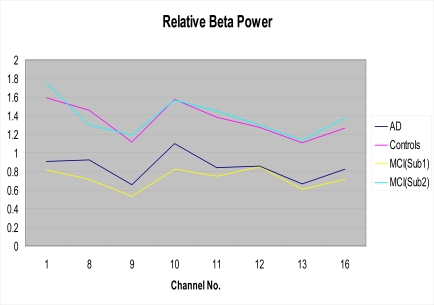
Relative beta power for the AD group, the controls and the MCI subgroups as a function of EEG channel
